# Meeting the Social Needs of Older Adults in Rural Areas

**DOI:** 10.1001/jamahealthforum.2020.1411

**Published:** 2020-11-02

**Authors:** Carrie Henning-Smith

**Affiliations:** Division of Health Policy and Management, University of Minnesota School of Public Health, Minneapolis.

Humans are built to live in community, and our health suffers when our social needs are not met.^1^ Yet, social isolation, defined as a lack of social contact, and loneliness, defined as a sense of being alone, are all too common. Early in 2020, research showed that more than 60% of all US adults are lonely. The coronavirus disease 2019 pandemic has only made matters worse, because people have distanced themselves from others and upended their daily routines. Given the associations that isolation and loneliness have with health and mortality risk,^1^ as well as avoidable health care costs, there is growing recognition of the importance of preventing and mitigating these issues and the critical role that health care can play in doing so.

Older adults face unique risks of social isolation and loneliness owing to a multitude of factors, including retirement, losses of spouses/partners and other loved ones, and changing health and functional status.^2^ Older adults are also the most likely age group to live alone, with rates especially high among older women. While living alone is not deterministic of loneliness, it is a risk factor.^3^

Residents of rural places also face distinctive risks for social isolation and loneliness.^4^ Residents of rural areas have multiple barriers to connecting with one another, including transportation challenges, built environments that are not always walkable or conducive to social interaction, more limited economic resources, less access to broadband Internet and cellular connectivity, and more restricted access to health care, including mental health care.^4,5^ Each of these is heightened for older adults in rural areas, who tend to be less mobile than their younger counterparts and more reliant on resources within their particular community.^5^ While older adults in rural areas report having larger social networks than older adults in urban areas, they also report higher levels of loneliness, indicating structural barriers to connecting.^4^

Challenges to addressing social isolation and loneliness among older adults in rural places exist alongside growing inequities in health between rural and urban areas.^6^ Rural residents have higher mortality and morbidity rates along nearly every measure.^6,7^ Notably, for issues of social isolation and loneliness, rural residents have higher rates of suicide and more limited access to mental and behavioral health services. Addressing social isolation and loneliness for older adults in rural communities must take this context into account.

There are a variety of ways in which to intervene on social isolation and loneliness for rural older adults. On an interpersonal level, judicious screening in a clinical setting is an important first step, but it cannot be the end goal. Any detection of isolation or loneliness should be followed up by well-coordinated referrals to programs that connect individuals, including friendly visitors, telephone calls, intergenerational programming, clubs and hobby groups, and so on. Many of these exist in rural communities already, although not all identify or market themselves as social connectedness programs, nor are they equally distributed across rural areas.

Meeting the interpersonal social needs of older adults in rural areas is intertwined with meeting any other needs. For example, home-delivered meal programs can help to provide food while also including social contact between the delivery driver and the recipient. Drivers providing home-delivered meals may be among the first to detect isolation, loneliness, or changes in mental health among older adults in rural areas. In some instances, these are the some of the only people with whom older adults who are isolated in rural areas have regular social contact.

Similarly, health care professionals should look to other community partners to collaborate on reducing isolation and loneliness and their deleterious effects. In rural areas, such partners could those in service roles, such as mail carriers, faith-based organizations, grocery store clerks, and bank tellers. These individuals may have more contact with older adults in rural locations than health care professionals, making them better positioned to recognize signs of isolation and loneliness and their health outcomes. Other potential partners include those in more traditional aging services, such as Area Agencies on Aging and local senior centers.

Importantly, however, preventing social isolation and loneliness among older adults in rural areas will not succeed without upstream interventions. As important as screening and interpersonal programming are, it is equally—if not more—important to address structural determinants of social contact for older adults in rural areas, such as transportation availability, access to broadband Internet and cellular connectivity, housing quality, and the built environment. These structural determinants influence how people go about their daily lives and the opportunities they have to interact with one another. They also affect whether older adults in rural locations are able to age in place where they have already established relationships. Each of these determinants is uniquely challenging to address in rural areas but not impossible, given broad support and political will. Achieving that requires recognition of health in all policies and the interconnectedness between health care, public health, and other sectors of society.

Successful models exist across the US of rural communities working creatively and resourcefully to improve social connectedness for older adults. These include interpersonal interventions, such as coffee groups for men in retirement and intergenerational programming. These models also include interventions further upstream, such as those that improve access to transportation, expand digital connectivity, and change the built environment of rural communities to be more accessible and inclusive. These structural interventions are essential to creating opportunities to spark social connections and should be a prerequisite for developing downstream interpersonal programming.

Former US Surgeon General Vivek Murthy, MD, MBA, describes loneliness as being similar to thirst. It’s the body’s way of telling a person that it needs something. We need each other. Older adults in rural areas are vital members of not only their communities but society, and there is a moral, public health, and economic imperative to support them in remaining socially connected. It is on us as researchers and practitioners in public health and health care to build on successful interventions, researching their efficacy and replicability; identify where gaps remain; advocate for evidence-based policy action; collaborate with other sectors; and, especially, listen to older adults in rural areas to better understand how they want to connect.
